# Optimizing DNA Extraction from Pediatric Stool for Diagnosis of Tuberculosis and Use in Next-Generation Sequencing Applications

**DOI:** 10.1128/spectrum.02269-22

**Published:** 2022-12-08

**Authors:** Tara E. Ness, Lennard Meiwes, Alexander Kay, Rojelio Mejia, Christoph Lange, Maha Farhat, Anna Mandalakas, Andrew DiNardo

**Affiliations:** a Division of Pediatric Infectious Diseases, Baylor College of Medicine, Texas Children’s Hospital, Houston, Texas, USA; b Global TB Program, Baylor College of Medicine, Texas Children’s Hospital, Houston, Texas, USA; c Respiratory Medicine and International Health, University of Lübeck, Lübeck, Germany; d Baylor Center of Excellence, Mbabane, Eswatini; e Division of Clinical Infectious Diseases, Research Center Borstel, Borstel, Germany; f German Center for Infection Research (DZIF), Partner Site Hamburg-Lübeck-Borstel-Riems, Borstel, Germany; g Harvard Medical School, Department of Biomedical Informatics, Boston, Massachusetts, USA; h Pulmonary and Critical Care, Massachusetts General Hospital, Boston, Massachusetts, USA; Quest Diagnostics

**Keywords:** DNA sequencing, extraction, *Mycobacterium tuberculosis*, stool

## Abstract

The WHO has endorsed the use of stool samples for diagnosis of tuberculosis (TB) in children, and targeted next-generation sequencing (tNGS) of stool has been shown to support diagnosis and provide information about drug susceptibility (DS). Optimizing extraction of DNA from stool for sequencing is critical to ensure high diagnostic sensitivity and accurate DS information. Human stool samples were spiked with various concentrations of Mycobacterium bovis bacillus Calmette-Guérin (BCG), and DNA was extracted from the samples using four different DNA extraction kits. Each sample was subjected to quantitative PCR for identifying Mycobacterium tuberculosis complex bacteria and underwent further analysis to assess the overall DNA yield, fragment length, and purity. This same process was performed with 10 pediatric participants diagnosed with pulmonary TB, and the samples underwent tNGS. The FastDNA spin kit for soil showed the best results on model samples spiked with known quantities of BCG, compared to the other extraction methods evaluated. For clinical samples, the FastDNA and PowerFecal Pro DNA (PowerFecal) kits both showed an increase in the overall DNA quantity, M. tuberculosis-specific DNA quantity, and successful targeted sequencing when testing was performed on stool samples, compared to the two other kits. Three samples extracted via PowerFecal and three samples extracted via FastDNA (from different patients) provided successful sequencing data, with an average depth of coverage of the *rpoB* region for FastDNA of 298 (range, 107 to 550) and for PowerFecal of 310 (range, 182 to 474), results that were comparable to one another (*P* = 0.946). The PowerFecal Pro and FastDNA spin kits were superior for extracting DNA from pediatric stool samples for tNGS.

**IMPORTANCE** This is the first study to compare Mycobacterium tuberculosis DNA extraction techniques from pediatric stool samples for use with sequencing technologies. It provides an important starting point for other researchers to isolate quality DNA for this purpose to further the field and to continue to optimize protocols and approaches.

## INTRODUCTION

In 2020, approximately 10 million people developed tuberculosis (TB), with around 1 million of them being children and 0.8 million people living with HIV (PLHIV) (1). PLHIV are at an increased risk of acquiring TB infection and have a >20-fold increased risk of TB disease progression compared to their HIV-uninfected peers (2). More than half of the cases in children are not diagnosed or reported, although around 20% of children under 14 years and over 40% of those younger than 5 years die from TB when not treated (3, 4). Both children and PLHIV frequently present with paucibacillary sputum, and children are often unable to produce sufficient sputum. This results in less frequent bacteriological confirmation of cases and less phenotypic drug susceptibility testing being available.

The presence of Mycobacterium tuberculosis in the gastrointestinal track of pulmonary TB patients has been known of for over a hundred years (5–8), and new molecular tools have improved the identification of M. tuberculosis DNA in stool samples down to a limit of less than 100 M. tuberculosis CFU per 50 mg of stool (9). Next-generation sequencing (NGS) is coming to the forefront of diagnostic tools to provide genotypic drug susceptibility information in patients with active TB and aid in designing TB treatment regimens (9). Recently, targeted NGS (tNGS) was used to predict drug susceptibility from stool samples (10).

DNA extraction methods from stool have been identified as a crucial step for downstream sequencing applications in microbiome studies (11–13). Obtaining quality DNA for sequencing is paramount to ensuring that the most accurate and comprehensive sequencing data are produced. If DNA is too fragmented during the extraction process or too much yield is lost during the purification steps, it can impact the accuracy and completeness of information generated by sequencing, while relying upon an extraction with a low yield of DNA can erroneously lead to sequencing results that suggest the presence of a homogenous strain population (11, 13).

Previous DNA extraction techniques on human stool have been investigated for use primarily in microbiome studies, with the goal to obtain as much bacterial (14) and fungal (15) DNA diversity as possible to define the microbial community composition. The largest study to date evaluated 21 different DNA extraction protocols and compared the percentage of samples with quantified DNA of less than 1.8 kb, the overall DNA quantity, and the bacterial community diversity to inform the International Human Microbiome Standards (IHMS) Consortium to recommend a universal DNA extraction protocol for stool for microbiome studies, referred to as Protocol Q (16). The protocol utilizes a QIAamp silica-based column after homogenization with zirconia beads and lysis buffer and several steps to remove PCR inhibitors (17). This method, while a helpful starting point for investigating DNA extraction techniques for M. tuberculosis DNA sequencing, is specifically geared toward human microbiome studies on short-read sequencing platforms, such as Illumina. Other studies have investigated kits such as the Qiagen Genomic-tip device, aimed at minimizing shearing for long-read metagenomic sequencing (18) but also extending the timeline (hours to days) from sample collection to final data output. Extraction of M. tuberculosis-specific DNA from stool for sequencing purposes to collect information on drug resistance has successfully been performed utilizing the FastDNA kit (10). Since different kits have not been directly compared for their ability to extract M. tuberculosis complex DNA from human stool and to preserve long M. tuberculosis DNA fragments for sequencing analyses, we compared four DNA extraction methods with Mycobacterium bovis BCG-spiked stools samples and stool from patients with pulmonary TB to determine the best method for further exploring this diagnostic modality, especially in those with paucibacillary disease, such as children and PLHIV.

## RESULTS

### Patient demographics for clinical samples.

All 10 participants had a positive quantitative PCR (qPCR) test for M. tuberculosis from their stool (3.32 to 1,736.11 fg/μL and cycle threshold [*C_T_*] values of 19.898 to 31.047). The patients’ ages ranged from 2 to 21 years, with a median age of 16.5 years (interquartile range [IQR], 10.75 to 20). Six male (four female) patients were included. All patients had had a GeneXpert test performed on their stool samples, which were positive in all but one patient, whose *C_T_* value was the highest (31.047).

### Quantity of overall DNA (Qubit).

**FIG 1:**
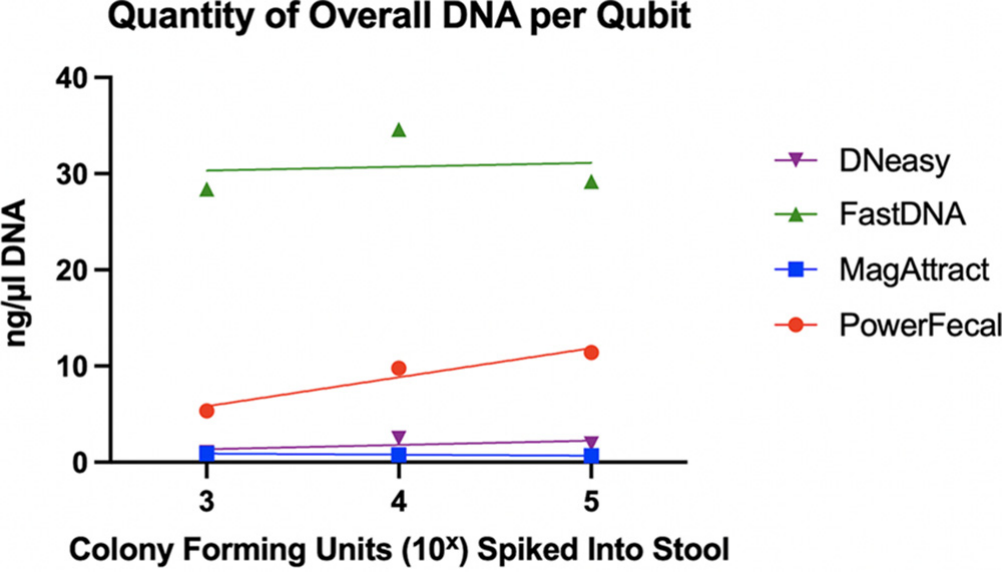
Quantity of overall DNA extracted from spiked stool samples as measured using a Qubit fluorometer.

**(i) BCG-spiked control samples.** The overall DNA quantity (not M. tuberculosis-specific) as measured using the Qubit fluorometer showed that the FastDNA kit (FastDNA) yielded the highest concentration of DNA (average, 30.7 ng/μL over three samples; range, 28.4 to 29.2 ng/μL), regardless of the initial CFU of BCG spiked into the stool sample; however, this result was not statistically significantly greater than that obtained using the PowerFecal kit (PowerFecal) (*P* = 0.25). The next-highest concentration of DNA was obtained using PowerFecal (average, 8.85 ng/μL; range, 5.36 to 11.40), followed by the DNeasy (average, 1.81 ng/μL; range, 1.02 to 2.49), and MagAttract (average, 0.797 ng/μL; range, 0.698 to 0.917) kits (Fig. 1).

**FIG 2:**
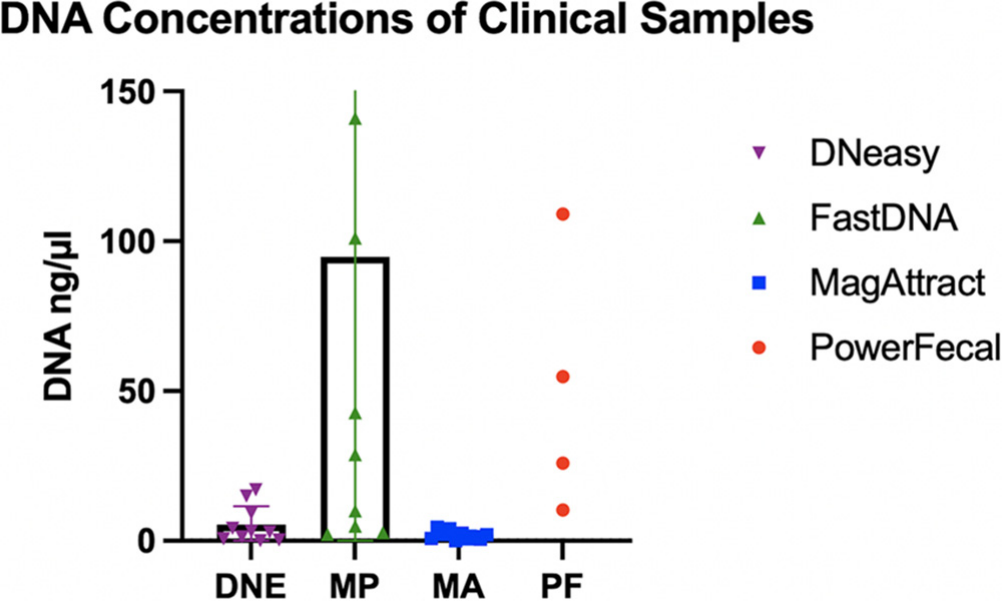
Quantity of overall DNA extracted from patient stool samples as measured using a Qubit fluorometer.

**(ii) Clinical samples.** The overall (not M. tuberculosis-specific) DNA quantity as measured using the Qubit fluorometer showed PowerFecal with the highest average DNA concentration (177.5 ng DNA/μL; range 10.3 to 644.0), followed by FastDNA (94.9 ng/μL; range, 2.20 to 455.0), DNeasy (5.4 ng/μL; range, 0.056 to 17.10), and MagAttract (1.9 ng/μL; range, 0.051 to 4.62). The difference in the total, non-M. tuberculosis-specific DNA concentration between FastDNA and PowerFecal was not statistically significant (*P* = 0.063); however, the difference between each of these two kits and DNeasy and MagAttract was statistically significant (*P* < 0.05 when comparing PowerFecal to both DNeasy and MagAttract, as well as when comparing FastDNA to DNeasy and MagAttract) (Fig. 2).

### Quantity of M. tuberculosis-specific DNA (qPCR).

**FIG 3:**
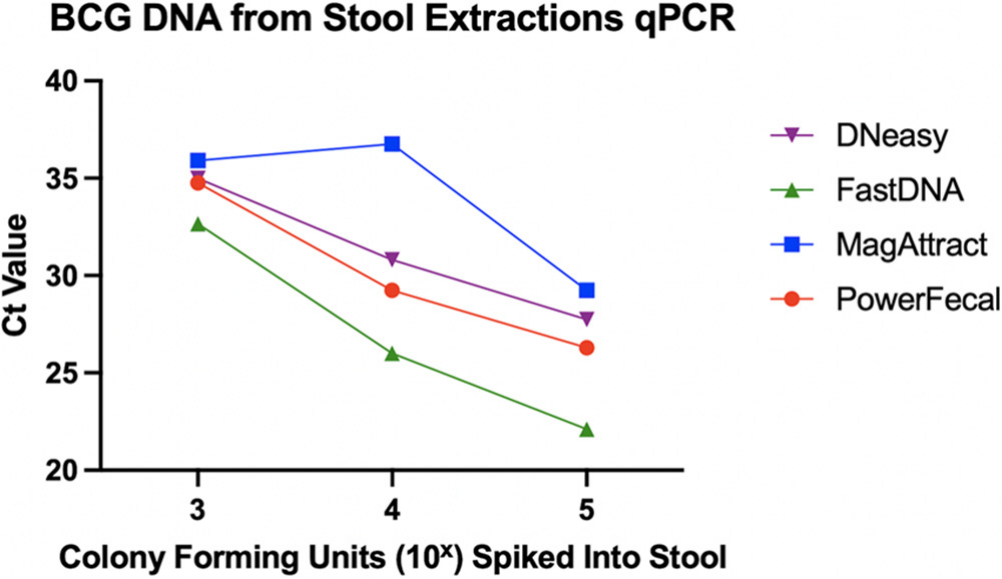
*C_T_* values from qPCR using known spiked dilutions of BCG into healthy human stool (paired *t* test, *P* = 0.0340).

**(i) BCG-spiked control samples.** Figure 3 shows the cycle threshold (*C_T_*) values in which M. tuberculosis DNA was amplified. All extraction kits showed an expected linear relationship between the amount of BCG spiked into the stool samples and the PCR *C_T_* values, with increasing CFU of BCG causing the M. tuberculosis DNA to amplify at a lower *C_T_* value. The FastDNA kit showed the lowest cycle threshold of amplification across all three dilutions (*P* = 0.0340, compared to PowerFecal), followed by PowerFecal, DNeasy, and then MagAttract.

**FIG 4:**
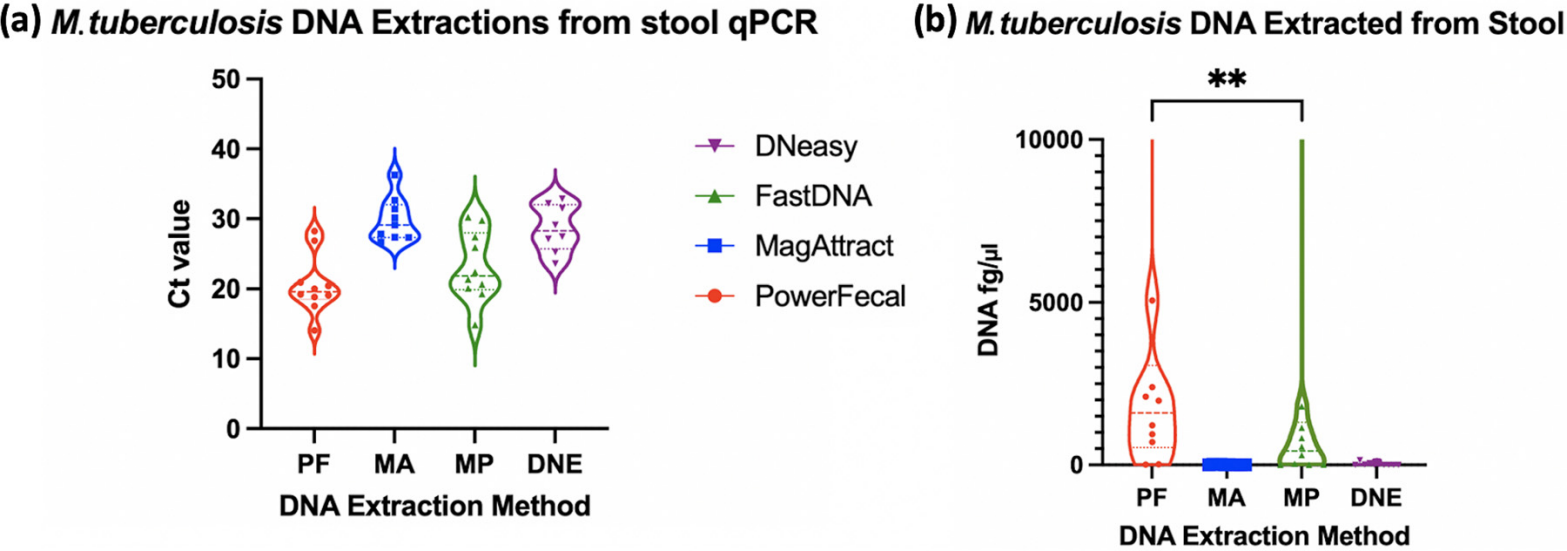
*C_T_* values of M. tuberculosis DNA from qPCR performed on the stool samples of patients diagnosed with pulmonary TB (a) and quantity of M. tuberculosis DNA extracted (b). Panel b shows significantly different (*P* = 0.0030) amounts of M. tuberculosis DNA extracted using the PowerFecal and FastDNA kits via Wilcoxon signed-rank test.

**(ii) Clinical samples.** Figure 4a and b depicts the *C_T_* values and calculated DNA amount for the extracted patient clinical stool samples, respectively. Fig. S1 in the supplemental material shows the M. tuberculosis *C_T_* values, with the clinical samples connected by a dashed line to show this on a per-sample basis. Overall, PowerFecal yielded the highest average amount (5,481.65 fg/μL) and median (1,602.29 fg/μL; IQR, 533.93 to 3,061.34 fg/μL) of extracted M. tuberculosis DNA, followed by FastDNA (average, 3,088.08 fg/μL; median, 430.81 fg/μL; IQR, 12.46 to 1,069.08 fg/μL), DNeasy (average, 24.55 fg/μL; median, 3.587 fg/μL; IQR, 0.48 to 17.39 fg/μL), and MagAttract (average, 8.12 fg/μL; median, 4.57 fg/μL; IQR, 0.93 to 16.28 fg/μL). The Wilcoxon signed-rank test showed a statistically significant difference between PowerFecal and FastDNA (*P* = 0.0020) when M. tuberculosis-specific DNA quantities were compared.

### Purity of DNA (NanoDrop).

**FIG 5:**
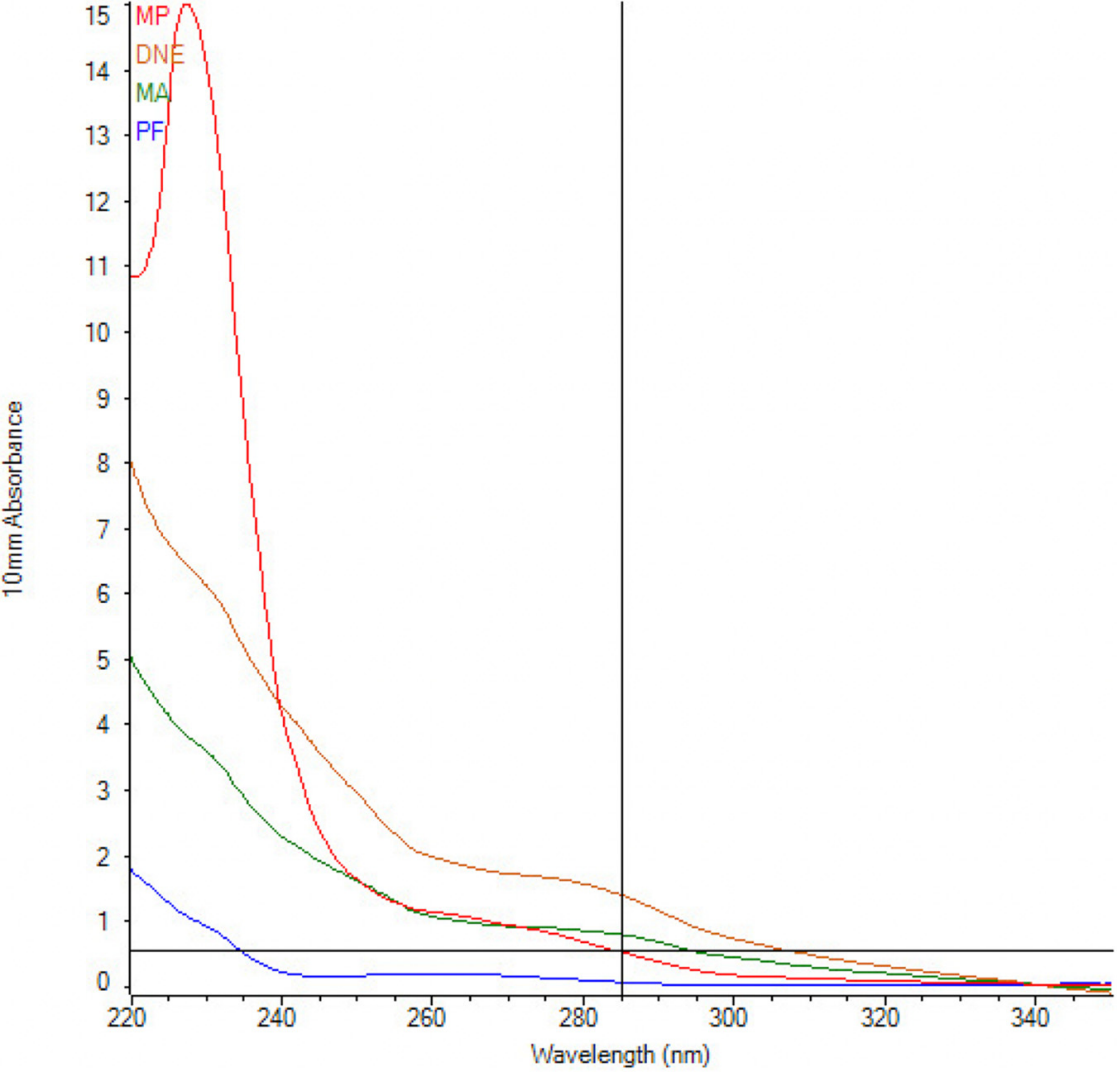
Overlay graph from the NanoDrop system showing significant contamination from all extraction kits in the 230 nm wavelength.

**(i) BCG-spiked control samples.** Table 1 and Fig. 5 summarize the NanoDrop spectrophotometer results for the 10^4^ CFU spiked sample for each of the DNA extraction methods. PowerFecal showed the highest *A*_260/280_ ratio (expected, ~1.8 for pure DNA), indicating the least contamination with protein or other reagents, followed by FastDNA, DNeasy, and then MagAttract. All samples showed the presence of contamination via the *A*_260/230_ ratio (expected, 2.0 to 2.2 for pure DNA), with DNeasy indicating the least, followed by MagAttract, PowerFecal, and then FastDNA.

**TABLE 1:** Spectrophotometric absorbance ratios obtained via the NanoDrop system^*a*^

Kit	*A*_260/280_	*A*_260/230_
PowerFecal	1.87	0.18
MagAttract	1.19	0.27
FastDNA	1.76	0.06
DNeasy	1.25	0.55

a The four different DNA extraction kits were used on known quantities of BCG (10^4^ CFU) spiked into stool.

**FIG 6:**
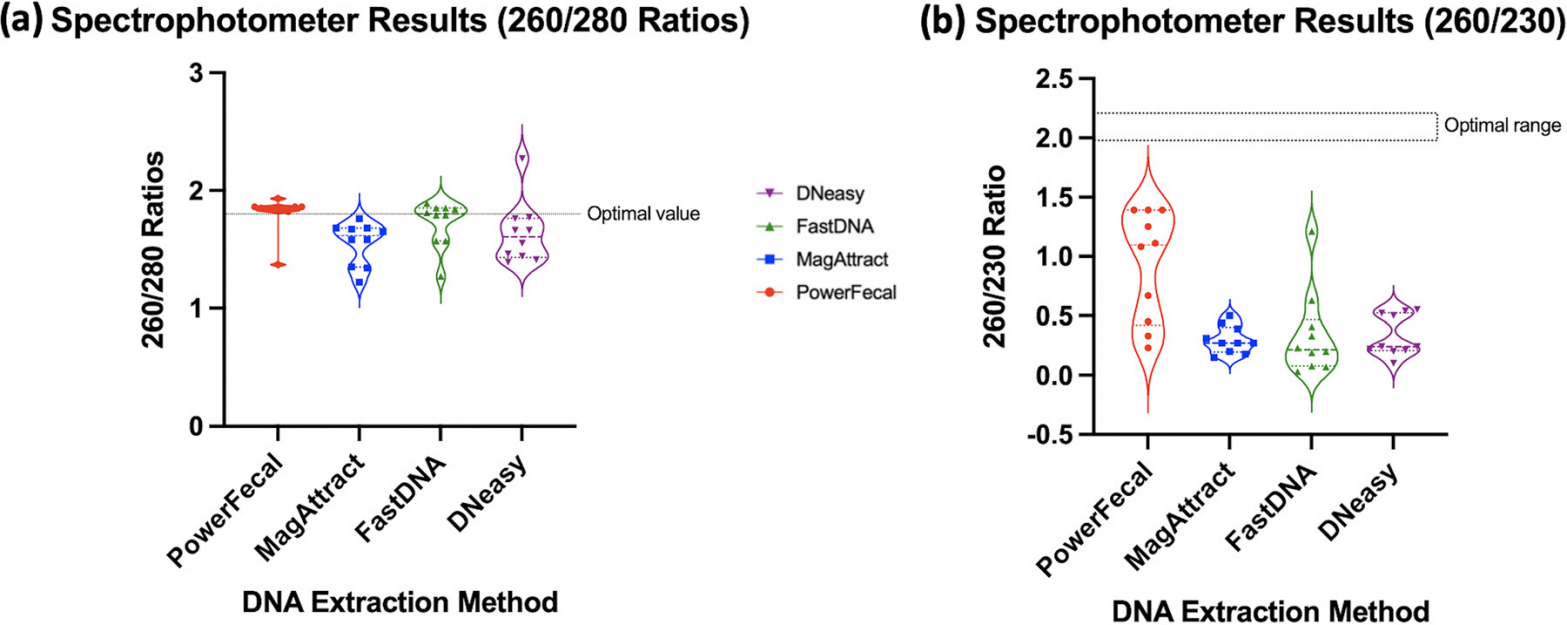
(a and b) Spectrophotometric ratios obtained from NanoDrop using the four different DNA extraction kits on 10 patient clinical samples. The optimal ratio is designated on each graph by horizontal dotted lines.

**(ii) Clinical samples.** Figure 6a and b shows the data obtained from the 10 patient clinical samples. Overall, the best *A*_260/280_ ratios were obtained using PowerFecal, followed by FastDNA. Increased wavelength absorption at 230 nm was observed with clinical replicates; however, PowerFecal had multiple samples for which this value was improved compared to the controlled BCG-spiking studies. Table 2 provides the median spectrophotometer results for the four DNA extraction methods.

**TABLE 2:** Spectrophotometric ratios obtained via the NanoDrop system^*a*^

Kit	*A*_260/280_	*A*_260/230_
PowerFecal	1.85	1.1
MagAttract	1.62	0.27
FastDNA	1.80	0.22
DNeasy	1.61	0.24

a Values were obtained using the four different DNA extraction kits on clinical samples; the values provided are medians.

### Fragment length (Agilent).

**FIG 7:**
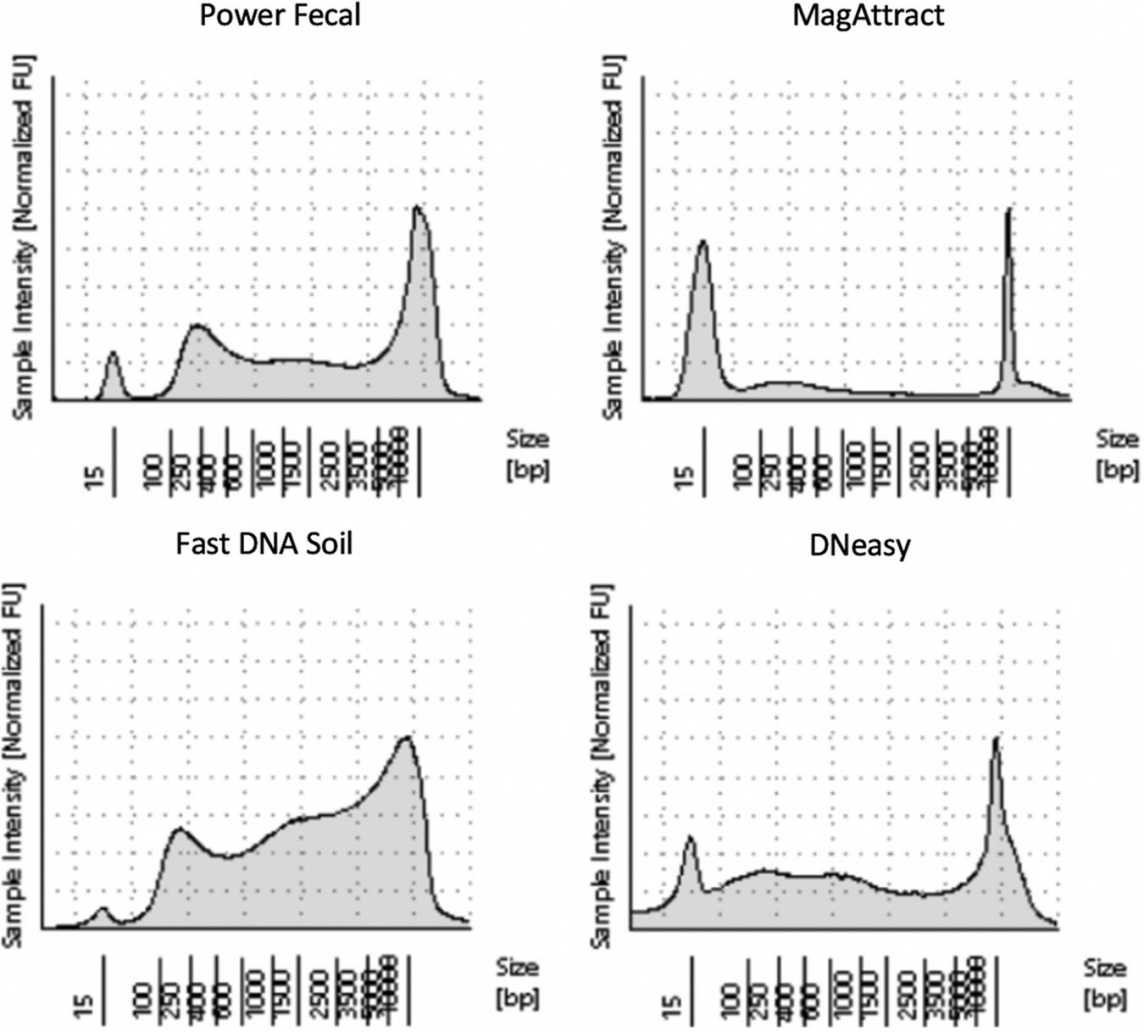
Visualization of the overall DNA fragment sizes from 15 to 10,000 bp using four different DNA extraction techniques as measured using Agilent TapeStation for BCG-spiked control samples at 10^4^ CFU.

**(i) BCG-spiked control samples.** Figure 7 shows the total DNA fragment sizes as measured by the Agilent TapeStation system, with the reference ladder peaks located at 15 bp and 10,000 bp. Although the system does not delineate M. tuberculosis-specific DNA (only total DNA), the results demonstrate that more than 50% of the total DNA fragments were greater than 1,500 bp using the FastDNA kit, with PowerFecal and DNeasy showing DNA fragment sizes distributed evenly from 100 to 10,000 bp, indicating that either the DNA was more fragmented during these extraction processes or the isolation procedure did not select for larger fragments compared to the Fast DNA kit.

### Success of amplification using Deeplex Myc-TB assay targeted PCR.

For a subset of five patients whose samples all showed successful qPCR amplification (median *C_T_*, 23.8 [range, 19.898 to 28.203]; average, 300.52 fg/μL [range, 51.02 to 546.16]) using each of the four DNA isolation kits (total, *n* = 20), the samples then underwent amplification using the Deeplex Myc-TB assay, which also included a positive and negative control. Qubit quantification and gel electrophoresis showed successful amplification of all five clinical samples isolated via PowerFecal and three clinical samples via FastDNA kit (the other kits did not have successful amplification, necessary for downstream sequencing). After barcoding and sequencing, six of the eight clinical samples showed sequencing read numbers (>4,000 total reads) indicating successful sequencing. This included three samples isolated via FastDNA and three via PowerFecal. Two samples (both isolated via PowerFecal) had sequencing reads of fewer than 500 total reads, with even fewer reads specific to M. tuberculosis. The *rpoB* region showed an average depth of coverage of 298 reads for MP Biomedicals FastDNA (range, 107 to 550 reads) and 310 reads for Power Fecal (range, 182 to 474 reads), which was not statistically different. Of note, all samples with a successful sequencing result had a *C_T_* value of less than 25 on qPCR. Table 3 summarizes the results of the five patients who underwent sequencing, including test results, the kits which provided successful sequencing, and the depth of coverage of the *rpoB* region. All patient sputum samples were positive via GeneXpert Ultra, with no rifampin resistance detected, and for all patients, M. tuberculosis grew in culture, with no rifampin resistance detected on phenotypic testing. All patient stool samples were positive via GeneXpert Ultra, with no rifampin resistance detected. There was no relationship between the AFB sputum smear result and the quantity of M. tuberculosis via GeneXpert in stool or sputum with successful sequencing or depth of coverage of the *rpoB* region.

**TABLE 3:** Comparison of results for five clinical patients

Patient	AFB smear result^*a*^	GeneXpert Ultra quantity for:^*b*^		Successful amplification and sequencing modality	Depth of *rpoB* region coverage (no. of reads)
		Sputum	Stool		
1	2+	Low	Low	None	NA
2	1+	High	Medium	FastDNA	550
3	2+	High	Low	FastDNA	182
				PowerFecal	107
4	None	Low	High	FastDNA	273
				PowerFecal	238
5	4+	Medium	Low	PowerFecal	474

a AFB, acid-fast bacillus.

b The GeneXpert quantity was based on *C_T_* ranges defined by the manufacturer as follows: high, <16; medium, 16 to 22; low, 22 to 28; very low, >28.

## DISCUSSION

Next-generation sequencing (NGS) enables rapid detection of M. tuberculosis drug resistance by identification of resistance-associated mutations detected in the mycobacterial genome, when performed on native specimens like sputum or stool (10, 19). We compared four commercially available DNA kits in order to find the optimal method for extraction of M. tuberculosis DNA from stool. The FastDNA and PowerFecal kits showed equal success at extracting DNA from stool from clinical patients and yielded acceptable sequencing reads using the Nanopore MinION, with targeted amplification using the Deeplex Myc-TB assay. The FastDNA kit performed optimally on controlled, simulated samples that used BCG as a model for comparing total extracted DNA, BCG-specific DNA, and DNA fragment lengths. Overall, the FastDNA kit was the best method for mycobacterial DNA extraction in order to achieve acceptable sequence coverage for drug resistance prediction of M. tuberculosis.

The FastDNA soil extraction kit, compared to three other DNA extraction kits, showed lower PCR cycle thresholds for isolating M. tuberculosis-specific DNA, higher overall DNA yield (though not statistically significant compared to PowerFecal), and the highest proportion of long DNA fragments. This test uses a silica-based spin filter method after lysing organisms and was designed for use on soil and other environmental samples. As this kit is designed to process samples with detritus that is large or irregularly sized, it likely performs well on stool samples that have a heterogenous texture and content. Interestingly, contrary to our *a priori* hypothesis, the DNA fragment sizes remained large (50% of fragment sizes greater than 1,000 bp), indicating that the homogenization step did not cause substantial DNA fragmentation. The FastDNA soil extraction kit was previously found to be superior to the FastDNA spin kit for feces (MP Biomedicals, Irvine CA) for extracting pathogen DNA from stool samples (20). These findings are likely due to the high-fiber dietary content in resource-limited countries and the FastDNA soil extraction kit’s ability to liberate DNA from samples with high fiber and vegetable material.

PowerFecal Pro, the next-highest performing kit on our BCG-spiked samples, uses a similar technology with a bead-beating homogenization step, followed by cell lysis, inhibitor removal, and DNA capture via a silica membrane spin column. Both PowerFecal and FastDNA performed well on patient clinical samples, with PowerFecal showing equivalent extraction of overall DNA but larger amounts of M. tuberculosis-specific DNA determined by qPCR. The difference observed between the controlled, spiked samples and the clinical samples may reflect the processing M. tuberculosis DNA goes through as it passes through the human intestinal tract prior to being excreted in feces, as this is something we are unable to simulate with our spiking experiments. It could also be due to sample processing, with the BCG “clumping” more in the simulated stool experiments. Both PowerFecal Pro and the FastDNA soil extraction kit showed successful amplicon-based sequencing of stool samples when qPCR *C_T_* values were less than 25.

Limitations of this evaluation need to be addressed. There are a plethora of DNA extraction techniques and kits available commercially that could be utilized for potential DNA extraction for sequencing. We chose kits based on their easy commercial availability, differing approaches to DNA isolation (bead size, homogenization, inhibitor removal, and column versus magnetic) and previously published successes of the kits for extraction of DNA from stool (10, 14, 21, 22). Investigation into other kits that employ other extraction methods but are also easily commercially available is warranted and may yield better results for sequencing. We analyzed 10 clinical patient samples with confirmed pulmonary tuberculosis from one geographic region and likely similar dietary habits, so our results are representative of a specific cohort. We tried to mitigate the effect of these variables by including the controlled spiking studies with BCG DNA spiked into human stool obtained from a healthy participant based in the United States under 5 years of age. Our *rpoB*-specific read counts are relatively low, which is likely due to the large amount of nontuberculous DNA found in stool samples and could be an area for future investigation and optimization. Lastly, our cases were largely smear positive, indicating a high bacillary burden; additional investigation of these methods on paucibacillary and sputum culture-negative participants should be undertaken next.

Two DNA extraction kits (FastDNA soil kit and PowerFecal Pro) showed successful qPCR of BCG in the controlled spiking studies and M. tuberculosis DNA in the clinical samples of patients who were diagnosed with confirmed pulmonary TB. These kits also were successful for downstream applications of tNGS, with successful amplicon amplification and sequencing on a portable sequencing device (Nanopore MinION). These methods are critical first steps to realizing the promise of stool-based NGS. By investigating and refining the methods for DNA extraction from human stool, we can optimize the success and quality of our downstream sequencing results to ensure that this method provides the best information for clinical decision-making for patients unable to provide alternative samples.

In conclusion, among four commercial assays, the FastDNA kit exhibited the best performance for mycobacterial DNA extraction for an NGS-based prediction of M. tuberculosis drug resistance.

## MATERIALS AND METHODS

Two approaches were utilized to investigate the best M. tuberculosis DNA extraction method for use with targeted next-generation sequencing (tNGS) and stool samples. The first method was to provide a tightly controlled, reproducible experiment that used known concentrations of DNA spiked into stool samples, and the second method was to investigate the application on clinical samples to determine the performance in a real-world setting. Four different DNA isolation kits were investigated (Table 4).

**TABLE 4:** DNA isolation kits and abbreviations

Kit name	Manufacturer	Abbreviation used
DNeasy blood and tissue kit (silica-based extraction)	Qiagen, Hilden, Germany	DNeasy
FastDNA kit (silica-based column, multiple bead sizes)	MP Biomedicals, Irvine, California, USA	FastDNA
MagAttract HMW kit (magnetic bead binding)^*a*^	Qiagen, Hilden, Germany	MagAttract
PowerFecal Pro DNA kit (silica-based column, small beads)	Qiagen, Hilden, Germany	PowerFecal

a HMW, high molecular weight.

Optimizations for the above protocols based on our sample type (stool) and other necessary modifications can be found in the supplemental materials. If not otherwise stated, extractions were performed according to the manufacturer’s instructions.

### Controlled BCG-spiking studies.

Mycobacterium bovis bacillus Calmette-Guérin (BCG) (2.6 × 10^6^ CFU) expressing click beetle red luciferase (CBRLuc) was supplied by J. Cirillo (Center for Airborne Pathogen Research and Tuberculosis Imaging, Bryan, TX, USA) and grown and stored at −80°C in frozen glycerol prior to use. Prior to extraction, the samples were spun down at 10,000 × *g* for 5 min. The supernatant was removed and discarded. The bacterial pellet was washed with 2× phosphate-buffered saline. The sample was then resuspended in 200 μL sodium phosphate buffer. Two small glass beads were added to each sample, which was vortexed well for 30 s, and the suspension was drawn up using a tuberculin syringe and 27-gauge needle placed on the syringe to dispense the suspension contents into 1,400 μL sodium phosphate buffer. The solution was vortexed, and 7 equal aliquots of 200 μL were created (approximately 3.7 × 10^5^ CFU per aliquot). Two 10-fold dilutions were created for six of the aliquots (roughly 3.7 × 10^4^ and 3.7 × 10^3^ CFU), and 50 mg of stool from a healthy human volunteer was spiked with each of these decreasing 10-fold dilutions. A control was prepared by adding 1,800 μL sodium phosphate buffer to the last 3.7 × 10^5^ CFU aliquot, which was aliquoted again into 200-μL aliquots (now roughly 3.7 × 10^4^ CFU). No stool was added to this aliquot, and it served as the pure BCG (no stool) control. All samples were vortexed for 15 s and frozen at −80°C for further use. Each set of samples (3.7 × 10^5^ BCG plus stool, 3.7 × 10^4^ BCG plus stool, 3.7 × 10^3^ BCG plus stool, and 3.7 × 10^4^ BCG-only control) was processed with one of the four DNA extraction kits. All extractions took place on the same day to ensure that no aspect of DNA degradation impacted the results. All extractions were eluted into 100-μL volumes of buffer, designated by the kit manufacturers to optimize consistency in concentrations. The CFU chosen (10^3^, 10^4^, 10^5^) correspond to approximately 50 to 10,000 fg/μL of M. tuberculosis DNA, quantified using our established primers and probes (23). There is limited information at present on the quantification of M. tuberculosis DNA in stool samples donated by patients with pulmonary tuberculosis, but in one previous study (10), values ranged from <1 fg/μL (37% with a positive stool qPCR) to >100 fg/μL (18% with a positive stool qPCR).

Quantitative PCR was performed on all samples (using previously described primers and probes) investigating the IS*6110* region (23). Subsequently, each sample underwent fluorometric quantitation using a Qubit fluorometer (Invitrogen, Waltham, MA, USA), electrophoresis using an Agilent TapeStation (Agilent, Santa Clara, CA, USA) to determine DNA fragment sizes, and spectrophotometric analysis using a NanoDrop spectrophotometer to determine the sample purity. All samples underwent investigation on the same day to ensure no variation in the instrument calibration or degradation of samples over time that would impact sample comparison.

### Application to clinical samples.

For the clinical samples, we utilized a randomly selected subset of samples (*n* = 10) obtained from a prospective study cohort in Eswatini of individuals under 21 years of age with confirmed pulmonary tuberculosis. Participants were considered to have confirmed TB if a respiratory specimen was positive by GeneXpert MTB/RIF Ultra (Cepheid, Sunnyvale, CA, USA) or liquid culture with *Mycobacteria* growth indicator tubes (MGIT; Becton, Dickinson, Franklin Lakes, NJ, USA) and confirmed with the TB Ag MPT64 rapid test (Standard Diagnostics, Yongin-si, Gyeonggi-do, Republic of Korea). Between 2014 and 2019, stool samples were obtained within 2 weeks of TB treatment initiation from individuals attending outpatient tuberculosis clinics at Mbabane Government Hospital, Baylor Children’s Foundation Clinic, in Mbabane and the Raleigh-Fitkin Memorial Hospital in Manzini. Stool samples were frozen within 12 h of collection at −80°C for storage. As part of this cohort, study data were captured by research assistants using uniform case report forms, which included age, gender, and HIV status. Approval was obtained from all necessary ethical bodies, including the Baylor College of Medicine Children’s Foundation Eswatini (IORG0006978) and the Eswatini National Human Health Research Review Board, Baylor College of Medicine Institutional Review Board (FWA-00000286) (Houston, TX, USA).

A single collection tube of stool from each clinical participant was allotted into four 50-mg aliquots. Each of these aliquots underwent processing and extraction as described above and in the supplemental materials per the manufacturers’ instructions. After extraction, the samples underwent fluorometric quantitation using a Qubit fluorometer and spectrophotometric analysis using a spectrophotometer (NanoDrop Technologies, Wilmington, DE, USA) to determine the sample purity. All samples underwent investigation on the same day to ensure no variation in the instrument calibration. Due to heterogeneity in the M. tuberculosis DNA concentration within a patient stool sample, the aliquots were compared as a group rather than on an individual basis.

A total of 10 patient stool samples, from which DNA was extracted using the four different DNA extraction kits in parallel, were analyzed. Quantitative PCR was performed on the stool samples of all patients to detect the presence of M. tuberculosis DNA using IS*6110* primers (23). The amount of DNA in samples with unknown quantities (patient samples) was calculated using the cycle threshold (*C_T_*) value against a five-point standard curve and expressed in femtograms per microliter.

A subset of patient stool samples which showed successful qPCR amplification using each of the four DNA extraction kits were subjected to further downstream analysis with amplicon-based PCR (*n* = 20). These patient samples were evaluated using the targeted amplification in the Deeplex Myc-TB assay (Genoscreen, Lille, France) and then purified using AMPure XP bead cleanup (Beckman Coulter, Indianapolis, IN, USA). Based on the results of Qubit DNA quantification and gel electrophoresis, the samples that were successfully amplified via the Deeplex assay were subsequently prepared for sequencing. Successful amplification was defined as the presence of bands observed on gel electrophoresis and DNA quantification results on Qubit greater than those of the negative control. Sequencing was performed on the Nanopore MinION Mk1B sequencing platform (Oxford Nanopore Technologies, Oxford, UK). This is an affordable and portable sequencing platform and was previously validated for use in conjunction with this system for targeted amplification (24), in addition to the Illumina sequencing platforms for which it was designed. A negative control was used in each experiment (BCG-spiked control samples and patient clinical samples) to ensure that no contamination or other variable was influencing the results.

### Library preparation and sequencing using the Nanopore MinION system.

Samples were chosen for sequencing based on successful amplification using the Deeplex Myc-TB assay. This included five samples that were extracted using the PowerFecal kit and three samples that were extracted using the FastDNA extraction kit.

Approximately 100 to 200 fmol of DNA underwent DNA end-prep, barcode ligation, and sequencing adaptor ligation and was loaded onto a SpotON flow cell (FLO-MIN106 version R9.4.1) on a Nanopore MinION Mk1B sequencing platform (Oxford Nanopore Technologies) per the manufacturer’s instructions. The ligation sequencing (LSK-109) and native barcoding (EXP-NBD104) kits were used, and a flow cell check was performed prior to sequencing with ample active pores confirmed.

The MinKNOW GUI version 5.1.8 platform was used for primary data analysis, which included the integrated Guppy software, which provides base calling and demultiplexing. Alignment for genus level identification was performed using minimap2 within the EPI2ME FastQ WIMP (What’s In My Pot) version 3.5.4 analysis workflow. Geneious Prime version 2022.0.2 was used for data visualization. Mycobacterium tuberculosis H37Rv (GenBank accession number NC_000962.3) was used as the reference sequence for alignment.

Statistical analysis was performed using Prism version 9. Analyses included the Mann-Whitney test and the Wilcoxon signed-rank test for pairwise comparisons. Statistical significance was set at less than 0.05.
