# Supplementary material for: Optimizing DNA Extraction from Pediatric Stool for Diagnosis of Tuberculosis and Use in Next-Generation Sequencing Applications
Source: Microbiol Spectr. 2022 Dec 8;11(1):e02269-22. doi: 10.1128/spectrum.02269-22 (PMC9927083; doi:10.1128/spectrum.02269-22)

Supplementary Text 1. Qiagen DNeasy Blood and Tissue Kit SOP (QIAGEN Catalog ID: 69504 or 69506)
Silica-based extraction technique.

Upon receipt of kit:
All reagents can be stored at room temperature.

Equipment used in this protocol in addition to kit reagents:
Centrifuge
Incubator (heat block is ideal but water bath can be used if heat block unavailable)
Hula mixer
Ethanol (100%, 200 proof)

Notes prior to starting procedure:
You may need to redissolve precipitates that have developed in Buffer ATL and AL prior to starting.

Preheat incubator to 56 degrees (water bath or heat block will work).

Have stool warmed up to room temperature prior to beginning.

Procedure:

1. Place 50 grams of stool into an Eppendorf tube and add 250 microliters of ATL buffer. Add 20 microliters of proteinase K and vortex. Let sit for 15 minutes at room temperature on Hula mixer.
2. Add 200 microliters buffer AL. Add 20 microliters proteinase K and 4 microliters of RNase A. Mix thoroughly by inversion and incubate for 90 minutes at 56 degrees Celsius.
3. After the incubation period, add 200 microliters pure ethanol and mix by vortexing.
4. Spin down the mixture at 14000 gs for 10 minutes.
5. Pipette the supernatant off into a DNeasy mini spin column inside a collection tube. Centrifuge at 6000 x g for one minute. Discard the flow through and collection tube.
6. Place the spin column into a new collection tube. Add 500 microliters Buffer AW1. Centrifue for 1 minute at 6000 x g for one minute. Discard the flow through.
7. Add 500 microliters buffer AW2 and centrifuge for 3 minutes at 20000 x g. Discard flow through and collection tube.
8. Place the spin column into a 1.5 or 2 mL microcentrifuge tube. Add 100 microliters of Buffer AE (elution fluid) and incubate for 5 minutes at room temperature.
9. Centrifuge for 1 min at 6000 x g.
10. Store DNA for downstream use.

Supplementary Text 2. Qiagen QIAamp PowerFecal Pro SOP (QIAGEN Catalog ID: 51804)
Silica based extraction method on a spin column. Eluted into non-EDTA solution for optimal sequencing performance.

Upon receipt of kit:
All reagents except for CD2 solution can be stored at room temperature. CD2 should be stored at 2-8 degrees Celsius.

Equipment used in this protocol in addition to kit reagents:
Vortex
Vortex adapter (or Disruptor Genie)
Microcentrifuge

Notes prior to starting procedure:
The extracted DNA from this protocol needs to be stored at negative 20 degrees (or negative 80 degrees) after extraction.

If you do not have a Vortex Adapter, you can use a Disruptor Genie (10 min at 2000 rpm)

Check if C1 solution has precipitated. If it has, heat to 60 degrees until the precipitate dissolves.

Take as many of the bead tubes out that you will use and spin down approximately 30 seconds at 15000 x g to ensure the beads have all settled at the bottom.

Procedure:

1. Take approximately 50 mg of stool (pea-sized amount) and place in a PowerBead Pro tube. Add 800 microliters of solution CD1 and briefly vortex to mix.
2. Place PowerBead Pro tubes on vortex adapter and vortex at maximum speed (2000 rpms) for 10 minutes (Disruptor Genie can be used for 10 minutes at 2000 rpms instead)
3. Centrifuge the PowerBead Pro Tubes at 15,000 x g (rcf) for 1 minute
4. Transfer the supernatant to a clean 2 mL microcentrifuge tube without disturbing the pellet. The supernatant will still have stool particles. Anticipate around 500-600 microliters.
5. Add 200 microliters of CD2 solution to each PowerBead Pro Tube and vortex for 5 seconds. Return CD2 solution to 2-8 degrees Celsius for storage.
6. Centrifuge at 15,000 x g (rcf) for 1 minute. Again avoiding the pellet, transfer up to 700 microliters of supernatant to a clean 2 mL microcentrifuge tube.
7. Add 600 microliters of Solution CD3 and vortex for 5 seconds.
8. Load 650 microliters of the solution onto an MB Spin Column and centrifuge for 15,000 x g (rcf) for 1 minute. Discard the flow through and repeat with the rest of the solution so that all solution has passed through the spin column.
9. Add 500 microliters of Solution EA (wash solution) to the spin column and centrifuge at 15,000 x g (rcf) for 1 minute. Discard the flow through.

10. Add 500 microliters of solution C5 to the MB spin column and centrifuge at 15,000 x g (rcf) for 1 minute. Discard the flow through and place the spin column into a new 2 mL Collection Tube.
11. Centrifuge at 16000 x g for two minutes. Discard the flow through and collection tube. Place the spin column into a 1.5 mL elution tube.
12. Add 100 microliters of solution C6 to the center of the white filter membrane, being careful not to pierce the membrane.
13. Centrifuge for 15,000 x g (rcf) for 1 minute. The eluted DNA is now ready for storage until needed for further applications. Again, the DNA should be stored at -20 degrees (or -80 degrees) as the elution solution does not contain EDTA.

What are the different reagents used?
Solution EA: Wash solution. Flammable. Older versions of this kit used Solution C4 instead.
Solution C6: 10 mM Tris. EDTA is avoided as this may impact PCR and sequencing reactions done downstream. Nuclease free water may be used instead.

Supplementary Text 3. Qiagen MagAttract SOP (QIAGEN Catalog ID: 67563)
Magnetic bead-based extraction method for optimizing DNA >150 kb. Gentle lysis approach aims to minimize DNA fragmentation for use in sequencing.

Upon receipt of kit:
All reagents can be stored at room temperature. Some reagents require ethanol to be added before use. Proteinase K can be stored at 2 to 8 degrees Celsius to prolong use.

Equipment used in this protocol in addition to kit reagents:
Magnetic rack
Incubator (heat block is ideal but water bath can be used if heat block unavailable)
Thermomixer (can substitute hula mixer or other mixer as needed)

Notes prior to starting procedure:
Samples will be incubated at 56 degrees Celsius for one hour (ok for this to go over but temperature is very important for optimal cell lysing). Before starting, pre-heat incubator.

Vortex the magnetic bead suspension prior to starting. You may also need to gently stir the mixture with a pipette tip to get a homogenous starting mixture to draw up from.

Procedure:

1. Take 50 mg of stool and place in a 2 mL Eppendorf tube and add 500 microliters of ATL (lysis solution). If needed, add more ATL solution to ensure stool is submerged. Add 20 microliters of Proteinase K. Mix by inverting the tubes several times.
2. Incubate the samples at 56 degrees Celsius for 1 hour, inverting tubes every 5-10 minutes.
3. Centrifuge the tube at 8000 rpm for 1 minute. Transfer the supernatant to a 2 mL Eppendorf tube taking care to avoid the pellet.
4. Add 4 microliters of RNase A to the sample and incubate at room temperature for 2 minutes.
5. Add 15 microliters of MagAttract Suspension G to the sample.
6. Add 280 microliters Buffer MB to sample. Place microcentrifuge sample into tube holder and place onto mixer. Rotate for 3 minutes at 1400 rpms.
7. Place the tube onto the magnetic rack and wait until bead separation has occurred (approximately one minute). Remove the supernatant and discard. Avoid disturbing the magnetic bead pellet.
8. Add 700 microliters Buffer MW1 to the sample and place the tube holder onto the mixer. Mix for 1 minute at 1400 rpms.
9. Place the tubes on the magnetic rack and wait until separation has occurred (about one minute). Remove the supernatant and discard.
10. Repeat steps 8 and 9.
11. Add 700 microliters Buffer PE to the sample and place the tube holder onto the mixer. Mix for 1 minute at 1400 rpms.
12. Place the tubes on the magnetic rack and wait until separation has occurred (about one minute). Remove the supernatant and discard.

13. Repeat steps 11 and 12. Using a small pipette, try and pipette off any leftover supernatant, being careful to not disrupt the magnetic bead pellet.
14. While keeping the tubes on the magnetic rack so the magnetic bead pellet is stationary, "rinse" the beads carefully with 700 microliters nuclease free water. This is best done by pipetting slowly onto the opposite wall of the beads. Remove the water and discard.
15. Repeat step 14 with an additional 700 microliters of nuclease free water and remove as much water as possible using a smaller pipette, being careful not to disrupt the magnetic bead pellet.
16. Remove the tubes from the magnetic rack and add 100 microliters of Buffer AE (elution buffer). Mix at room temperature for 3 minutes at 1400 rpms.
17. Place the tubes back on the magnetic rack and wait until bead separation is complete (about one minute). Transfer the supernatant to a new DNA tube and store for later applications.

Supplementary Text 4. MP FastDNA Extraction Kit for Soil SOP

DNA is extracted from stool using soil- DNA extraction kits (MP FAST DNA), which rid the sample of fibrous organic material.

1. Label all tubes ahead of time. Each sample will require:
    a. ONE LYSIS MATRIX E TUBE
    b. ONE 2ML TUBE (STEP 7)
    c. TWO MORE 2ML TUBES (STEP 11)
    d. FINAL 2ML CATCH TUBE (STEP 24)
    e. ONE SPIN FILTER
2. Prep Sodium Phosphate Buffer with Internal Control (add 2ul IC)
    a. Dilute Internal Control in Sodium Phosphate Buffer to final concentration of $1:10^{10}$ dilution
    b. For example, if 10^8 stock of IC, add 0.5mL of IC to 60mL of Sodium Phosphate Buffer
    c. Keep Sodium Phosphate Buffer with IC in 4^0 C fridge after spiking with IC
3. Add 978 μl Sodium Phosphate buffer to Lysing Matrix E tube.
    a. Use same tip to load all samples
4. Add 122 μl MT Buffer.
    a. Use same tip to load all samples
5. Add 50 mg of stool to each tube.
    a. Can use toothpick or new pipet tip to load
6. Homogenize (chooser 1)
    a. FastPrep Instrument 40 sec on speed setting 6.0
    b. Mini Beadbeater 2 min
    c. Disruptor Genie 5 min 3000 rpm
    d. R2D2 (Environmental:Soil): 30 seconds
7. Centrifuge 14,000 **g-force** for 10 min
8. Add 250 μl PPS to new 2 ml tube
    a. Use same tip
9. Transfer supernatant from (Step 6) into 2 ml tube approximately 800ul (Step 7) and invert 10 times by hand
    a. Centrifuge 14,000 **g-force** for 10 min
10. In 2 new tubes, add 500 μl Binding matrix
    a. Re-suspend binding matrix vigorously and pipet from bottom of bottle
    b. Use same tip to load binding matrix
    c. Mix binding matrix every 10 tubes
11. Add supernatant from step 9 (approximately 600 μl) to each tube w Binding Matrix
    a. Volumes may vary, divide the supernatant equally
    b. Use same tip per sample, but change between numbers
12. Invert for 2 min
    a. Can store at 4 C for 1 hour at this step
13. Let sit for 5 min
    a. Room temperature

14. Remove& discard almost all supernatant from each tube.
    a. Remove at angle, careful not to disrupt matrix
    b. Remove enough supernatant leaving approximately 1 mm of volume behind
    c. Use same tip per sample, but change between numbers
15. Remove entire binding matrix & transfer to spin filter tube
    a. Use same tip from Step 14
16. Centrifuge 14,000 **g-force** for 2 min (or 14000 RCF)
    a. Depending on consistency of sample, more spins maybe needed to elute fluids
17. Empty catch tube and discard fluid
18. Add 500 μl of PREPARED SEWS-M to matrix and mix with gentle pipetting
    a. Careful not to pierce membrane at bottom of filter tube
19. Centrifuge 14,000 g for 2 min
20. Empty catch tube and discard fluid
21. Centrifuge 14,000 g for 2 min
    a. This helps dry the matrix
22. Replace tube with final catch tube
23. Air dry the filter for 5 min at room temp
    a. Leave lid open
24. Add 100 μl of DES and mix matrix with gentle STIRRING
25. Centrifuge 14,000 g for 2 min
26. Store sample at -80 C for long term storage

Supplementary Figure 1

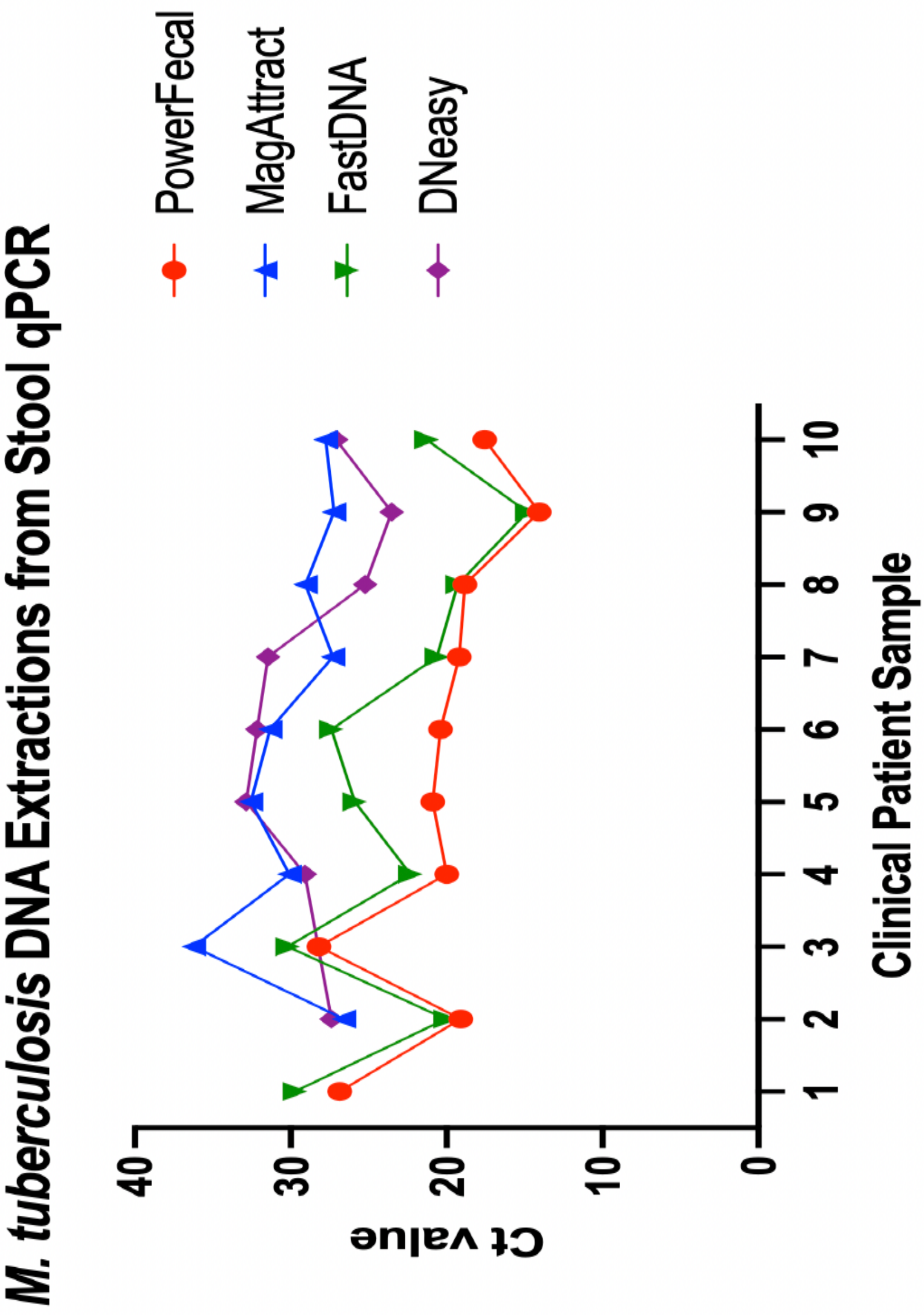

Supplement: Supplemental file 1 — Supplemental material. Download spectrum.02269-22-s0001.pdf, PDF file, 1.1 MB [file spectrum.02269-22-s0001.pdf]
